# Adapting to change: Exploring reverse migration as a coping strategy among internal migrants in Bangladesh

**DOI:** 10.1016/j.heliyon.2023.e19479

**Published:** 2023-08-26

**Authors:** Avijit Saha, Arpita Dutta, Minhazur Rahman Rezvi, Ridwan Islam Sifat, Nayeem Sultana, Nuruzzaman, Mehedi Hasan

**Affiliations:** a Department of Development Studies, Faculty of Arts and Social Sciences, Bangladesh University of Professionals, Dhaka, 1216, Bangladesh; b Department of Development Studies, Faculty of Social Sciences, University of Dhaka, Dhaka, 1000, Bangladesh; c School of Public Policy, University of Maryland, Baltimore County, Baltimore, MD, 21250, USA; d Department of Environmental Science and Technology, Jashore University of Science and Technology, Jashore, 7408, Bangladesh

**Keywords:** Internal migration, Reverse migration, COVID-19, Preparedness, Coping strategies

## Abstract

The COVID-19 pandemic has slowed down economic growth and disrupted labor markets throughout the world, including Bangladesh. A significant proportion of people lost income sources in the formal and informal sectors, triggering them to return to villages, and the transition introduces us to the new phenomenon known as “reverse migration”. This study explores and synthesizes the COVID-19 induced changing patterns of migration and returnees' coping strategies based on their level of preparedness as well as resource mobilization. A mixed-method research approach was applied to conduct the research. The study area was Rangpur (Pirganj, Taraganj, and Kaunia). For collecting primary data, semi-structured survey questionnaires were used and conducted 84 field survey data, 12 Focus Group Discussions (FGDs), 6 In-Depth Interviews (IDIs), 2 Key Informant Interviews (KIIs), and participant observations. Descriptive statistics and thematic analysis with the assistance of NVivo software were used to present the findings of this study. The findings of the study revealed that the COVID-19 pandemic fueled informal job holders’ returning to their homeland due to a low level of preparedness and mobilized resources. The study found that most respondents were in severe level unemployment. As a result, a lack of physical assets, they could not start new income-generating ventures and encountered food insecurity due to unexpected price hikes. The alarming result indicates that internal reverse migration is gendered, and the adverse impact is more prevalent among female migrants rather than male migrants. Along with the governmental organizations, the highlights of this study would be essential for non-governmental organizations and development practitioners.

## 1 Introduction

Migration, as an integral component of both regional and international processes, has a substantial impact on the contemporary world and the spatial mobility of societies, including family, social bonding, economy, politics, culture, and communication [1]. In last few decades, Bangladesh has witnessed the dynamic movements of its citizens. The movement of the migrants are visualized in both internal and external spheres, which grabbed the attention of the social think-tanks. Likewise, during COVID-19, the practice of reverse migration in Bangladesh has received significant attention and has become both a major concern and the subject of heated public debate.

Bangladesh is experiencing a growing trend of rural residents moving from their villages to metropolitan centers. This phenomenon is a result of the pull factor including better economic possibilities and push factors indicating the difficulties that rural residents confront, including an elevated risk of discomfort, rodenticides, illnesses, and erratic rainfall patterns. Consequently, rural families find themselves without the necessary resources to adequately withstand against these shocks. Furthermore, current intra-village informal insurance systems sometimes fall short of providing adequate protection, while formal insurance choices remain out of reach. They frequently opt to transfer migrants to metropolitan regions as a means of diversifying their income sources. By relying on the remittances sent by these migrants, rural families are able to fulfill their basic needs and sustain their livelihoods. McPherson reported urban resettlement of 1,500 families to cities, mostly Dhaka in search of economic opportunities and to earn bread and butter for their families [2].

The aforementioned economic potential for rural households experienced a significant setback when the COVID-19 pandemic unleashed its fierce splash globally. Mukhra et al. [3] documented in their research that despite the uncertain origins of the SARS-CoV2 disease, it was declared a global pandemic within a short span of two months since the first reported case. As a result, it has gained widespread recognition as a critical global health emergency. Governments around the globe-imposed lockdowns to halt the spread of the pandemic, which extended for several months. The consequences of subsequent limitations exacerbated internal migrants' multifaceted vulnerability in a number of developing countries [3].

In an effort to mitigate the spread of the pandemic during its initial stages, the government of Bangladesh implemented a series of stringent measures commonly referred to as “lockdown-type” measures in mid-March. However, these measures had an adverse effect on the economy, bringing it to a standstill and inflicting considerable financial hardships upon the general public and businesses, as highlighted by Rahman et al. [4] in their research.

Disadvantaged individuals and laborers, faced with limited job prospects, reduced opportunities, the imminent fear of an uncertain future, and financial upheaval brought about by the COVID-19 lockdown, initiated a significant wave of repatriation to their home countries [5,6]. Workers' real income has plummeted as labor demand has decreased and inflation has slowed. Earnings dipped by 37% overall, but by 42% in Dhaka and 33% in Chattogram [7].

As stated in a World Bank estimation, the COVID-19 pandemic has affected the lives of over 40 million internal migrants. Additionally, a substantial migration flow of approximately six hundred thousand individuals has swiftly transpired from urban to rural regions of origin in a matter of days. On this premise, Dandekar & Ghai [6] found that the unusual reverse nature of internal migration estimates is two and a half times higher than determines of foreign migrants. BRAC Institute of Governance and Development (BIGD) along with Power and Participation Research Center (PPRC) found in their studies that, during April 2019, approximately six percent of low-income individuals left Dhaka to return to their country homes. This percentage of returns notably increased to 15.64% by June [8]. Several studies in Bangladesh [4] evaluated the immediate effects on rural livelihoods, income loss, and food security, and it is evident when the study [9] claims that the Rangpur division has been designated as having the biggest number of poor people during COVID-19 in Bangladesh. Several studies have undertaken to evaluate the socio-economic status of migrant workers upon their return COVID-19 pandemic considering COVID-19 pandemic context. This study aims to contribute a distinct perspective from previous research, enabling relevant stakeholders and policymakers to gain a more comprehensive understanding of the essential support measures needed for returnee migrants. Additionally, the study enables academics and practitioners to have a more detailed understanding of the socioeconomic position of returnee migrants in order to make migration-related decisions during pandemics or crises.

The key objective of this study is to explore the patterns and strategies of reverse movement among the internal migrants of Rangpur district during the COVID-19 Pandemic. Similarly, inspection and exploration of returnees are motivating factors behind internal migration, general perception regarding the pandemic, level of preparedness based on resource mobilization, and future trend of migration.

## 2 Conceptual Framework

In recent years, migration has been fueled by war and bloodshed, economic and political uncertainty, and unpredictable climatic variations. The COVID-19 pandemic and its effects on the international and national economy have significantly changed the shape, pattern, and manner of migration [10]. Withstanding with migration, reverse migration has occurred both within and externally in Bangladesh since January 2020 [11].

International Organization for Migration [12] defined Reverse migration as “the act or process of going back to the point of departure; the returning of people to their origin or place of habitual residence after spending some time at another place.” It may involve a voluntary return or a forcible migration. The regular and repetitive movements between the origin and destination define seasonal, transitory, and circular migrations, whereas Return migration occurs after a longer period of unbroken travel, as remarked by Constant & Zimmermann [13]. Circular or chain migration is a prominent phenomenon observed in developing countries indicated by Rahman Bhuyan et al. [14] where migrants facing financial hardships and disillusionment may opt to return to their rural villages, thus conceptualized reverse migration from urban to rural areas. Scientific findings suggested downturn of economy and lack of employability in the rural homeland leads to normal migration. Whereas the relevance between the origin and destination areas made the informal workers to inclined to the totally opposite direction [15].

Numerous migrants have been adversely affected by the COVID-19 epidemic. Rahman et al. [16] in their study found a representative percentage of reverse migration during periods of shrinking livelihood possibilities, and significant reverse migration (urban to rural) has occurred in Bangladesh as a result of the epidemic [16]. In developing countries, Dandekar & Ghai [6] suggested through their scientific study that the COVID-19 pandemic has triggered a massive reverse migration. Significant numbers of domestic migrant workers, especially those in the informal economy, found themselves in a precarious position as a result of the COVID-19 pandemic. These individuals are stranded and unable to return to their respective residences because they cannot access public transportation and are restricted in their movements. A detailed study on India and Latin American countries by Mukhra et al. [3] pointed the crisis been resulted in a chaotic and traumatic process of mass return for internal migrants. A study by Chatterjee et al. [17] highlighted the three major reasons of reverse migration in the largest country of the subcontinent, India are (1) unemployment, (2) lack of food, and (3) scarce savings. In India, the COVID-19 pandemic caused the loss of 120 million jobs [17]. The COVID-19's lockdown resulted in extreme unemployment. Informal workers such as auto drivers, hawkers, and vendors had lost their income sources [6]. As a result, unemployment, poverty, and hunger, as well as fear of a pandemic, have increased people more desperate to return to their villages [3]. The COVID-19 pandemic has wrought devastation on the global economy, resulting in business interruptions and shutdowns due to social distancing measures.

Similarly, COVID-19 not only created a public health crisis but also caused a debilitating economic threat to Bangladesh. Income erosion and a lack of livelihood options exacerbated by the prolonged lockdown have forced people to leave the rural areas [16]. As reported in a survey conducted by the Bangladesh Institute of Development Studies (2020), approximately 13% of the population in the country has lost their jobs as a result of the COVID-19 outbreak and its detrimental impact on salary, turnovers, employment and expenditures of people, particularly in informal income groups [18]. Other than the newly unemployed, a large portion of the population has experienced a drastic drop in income [18]. BRAC [19] showed that almost 95% of people in the country have experienced a significant decline in their income. Informal and women-centric occupations had a greater loss of livelihood than formal sector occupations. Unskilled labor, transportation employees, and small enterprises all witnessed their earnings shrink by over 50% [16].

**Fig. 1:**
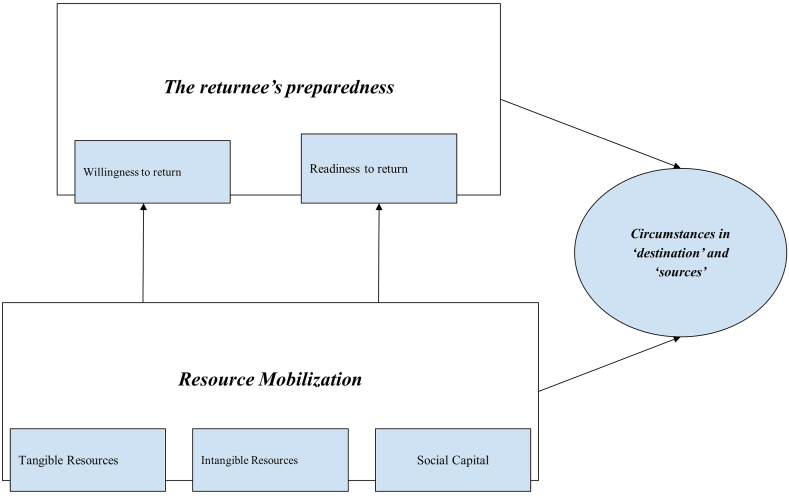
Conceptual Framework: Returnee's level of Preparedness and Resource Mobilization (Source: authors analysis based on Cassarino's study [20]).

**Fig. 2:**
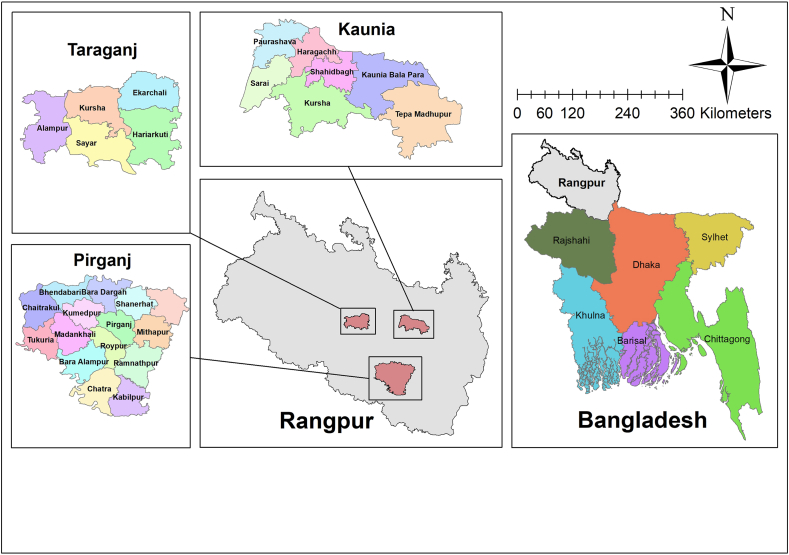
Map of the Study Area (Prepared using ArcGIS).

During COVID-19, unskilled labor, transport workers, and informal laborers have experienced the loss of income sources, and inclinations of preparedness of reverse migration have been visible. There are several factors that force or influence people to their areas of origin. As Cassarino [20] stated that returnees’ preparation for their return depends on motivation to return and also patterns of resource mobilization (Fig. 1) (see Fig. 2).

Following Fig. 1, preparedness for return (i.e., willingness to return and readiness to return) is determined by resource mobilization. Cassarino [20] defines resources in three types such tangible resources, intangible resources, and social capital. Physical capital, financial capital and natural capital are tangible resources. Contrast, human capital, which consists of labor's skills, experience, knowledge, creativity, and resourcefulness, is an example of an intangible resource. While social capital refers to the character of interpersonal relationships within a society. Migrants' patterns of resource mobilization vary based on their experiences and social backgrounds. The prevalence of reverse migration intentions becomes more apparent when migrants recognize that they have accumulated adequate levels of tangible, intangible, and social capital to ensure their livelihoods in their village. As noted by Cassarino [20], returnee preparedness is characterized by a voluntary and informed decision, supported by the effective mobilization of private resources and the acquisition of relevant knowledge. The study employed descriptive analysis, focusing on mixed method data collection techniques.

## 3 Methodology

### 3.1 Research method

This study opted for this approach to explore the pattern, perception, and investigation of the challenges and future trends of reverse migration due to COVID-19. Both approaches provide better understanding of research problems in an individual investigation [21]. The method was chosen because it gave respondents the opportunity to communicate their views through their perspective rather than requiring them to respond. Furthermore, it assists the qualitative comprehension of complex incidents by providing an explanation of the phenomena applying numerical data, data visualization, and basic statistical analysis.

### 3.2 Sample size and sampling technique

The purpose of this research is to investigate the pattern of internal-reverse migration; hence the internal returnees were chosen. The primary data was acquired from 84 migrants from the Rangpur district via field survey using semi-structural questions. Non-probability purposive sampling was used to select study participants. The research focused on internal reverse migrants of Rangpur district.

### 3.3 Study area

This study was conducted in Pirganj, Taraganj, and Kaunia-three Upazilas of Rangpur district. The study area was located at the East, West, and Southern corners of the district. The study area was selected on the basis of its poor income sources and economy, the low quality of public services, and the presence of challenging environmental conditions.

### 3.4 Tools of data collection

Before beginning data collecting for this study, inclusion and exclusion criteria had to be established. Respondents were selected only if they fitted the inclusion and exclusion criteria. The following are the two standards that have been established.

1. The criteria for inclusion were based on participants who had returned to their village from different metropolitan cities in Bangladesh during the lockdown.
2. Respondents who worked and remitted tangible or intangible resources to their families.

Both primary and secondary data were gathered to accomplish the research objective. Secondary data were gathered from publicly available sources, such as books, papers, reports, and web-based research projects and reports. Secondary data were analyzed in terms of theme, pattern, and perspective and also were examined to compare and contrast the information by linking them with the relevant literature. The primary data was collected through field-survey following semi-structural questionnaires and the study was also used FGDs, IDIs, and KIIs as research tools for collecting the primary data, those were as follows:

#### 3.4.1 Focus group discussion (FGD)

For in-depth conversation on a certain issue, focus groups (FGD) bring together people with similar experiences and perspectives. Participants were chosen using non-probabilistic, purposive sampling based on their availability during the data collection period (August 2020 to November 2020). The participants were guided by a facilitator who introduced discussion topics. Additionally, the facilitator provided each participant with a semi-structured questionnaire. The study consisted of twelve FGDs, with an average of seven participants per session. During the COVID-19 pandemic, FGDs were administered in an open field while maintaining social distance.

#### 3.4.2 In-depth interviews (IDIs)

Using non-probability purposive sampling, we also conducted six in-depth interviews (IDIs). We conducted three field interviews to enhance the design of the questionnaire. The researcher modified the interview questionnaire to suit the conditions. Changing interview criteria after the initial interview is common in qualitative research [22,23]. Although we ensured a high level of confidentiality, three potential participants declined to participate in the study due to privacy concerns. Following open-ended questionnaires, in-depth interviews ranging from 33 to 60 min per interviewee were conducted. To protect the participants' privacy, their names will not be included in the survey results. Due to the unwillingness of some participants to be documented, the interviews were written down rather than recorded on a mobile device. Other researchers transcribed and evaluated the transcripts of the interviews to ensure accuracy and reliability.

#### 3.4.3 Key informant interviews (KII)

A key informant is someone who can be called upon as an expert source of information [24]. For this, the technique was selected to collect the necessary information. The respondents were the project manager of the “Empowering the Poor through Federations” project of Rangpur Dinajpur Rural Service (RDRS) Bangladesh and an Upazila Health and Family Planning Officer, interviewed as a part of KII. The interviews were conducted over the phone. All the interviews were conducted with informed consent. Personal details of the interviewees were not used in the research.

### 3.5 Quality assurance

Cross-checking was performed on the quantitative data collected from the survey, followed by data cleaning and editing to ensure that there were no errors in the responses and the information provided. The facilitator input the data into the KoBo Toolbox and then used Microsoft Excel to clean, edit, and analyze it because the software provides the flexibility to clean and modify the collected data. NVivo 12 by QSR International was utilized for organizing data and analysis in qualitative research.

NVivo is beneficial for analyzing large amounts of text data because it provides a more in-depth analysis and better data analysis [23,25]. The most effective software for coding, classification, and theme design is NVivo [26]. After returning from the field, the data collection was carefully organized and compiled using a wide range of approaches.

The field-level research assistant took notes during the interviews and FGDs, typed up the notes, and used “verbatim” quotes from the interviewees' speeches. We utilized triangulation techniques to ensure the accuracy, validity, and reliability of our data. In this study, the investigator triangulation and data source triangulation techniques were utilized because they were essential for reducing bias in data collection, reporting, and analysis [27]. In order to adhere to investigator triangulation guidelines, the researchers frequently visited the field to investigate while collecting data without prior consultation. This method of data acquisition guaranteed exceptional data validation. To accomplish a high-quality study, we simultaneously collected data through three channels, including IDIs, FGDs, participant observation, and KIIs, as a data source triangulation process.

### 3.6 Ethical consideration

All the interviews have been carried out with informed written consent. All procedures performed in studies involving human participants followed the ethical standards with the 1964 Helsinki declaration and its later amendments or comparable ethical standards.

## 4 Result

The findings were tabulated and shown to illustrate the information in line with the outcomes outlined in the study. The in-depth interviews (IDIs) of respondents and key informant interviews (KIIs) were also located in the same area of the results, alongside analysis, verification, and transcription (see Table 1).

### 4.1 Demographic information of the respondents

Table 2 presents a summary of the respondents' background characteristics. The sample represented a higher portion (36.9%) of participants (35–45 years) who had experienced reverse migration. The age group (25–35 years) was not so far behind (34.52%) in terms of that. The other two age groups (15–25 years and >45 years) were far behind (10.71% and 17.86%, respectively) of the first two groups. Male respondents (60.71%) outnumbered female respondents (39.29%). The majority of respondents (61.90%) were married, with just a small portion (9.52%) being unmarried. Notably, a large percentage (19.04%) had been widowed, and 7.14% had been abandoned by their spouses. Additionally, the evidence from Table 2 suggested respondents had none to negligible educational background, with 30.95% being without formal education and 36.90% having primary education. The former evidence justified the background of having a larger family size (21%), having more than 4 members in the family with a high number of individuals, and 36.90% with a below-average monthly income (5000–10,000 BDT or $58–117 USD).

**Table 1:** Summary of Preparedness Level connecting to Pre-return and Post Return Condition.

Level of preparedness	Pre-return condition			Post return condition
	Motivation	Resources mobilization	Length of stay	Reintegration Process
High level of preparedness	● Migration objectives are reached ● Perceived positive changes in destination ● Economic improvements at home generates new opportunities	● Tangible resources (i. e., savings, land) ● Intangible resources (i.e., knowledge, skills, enterprise) ● Social capital (i.e., relationship, contacts)	On average, from 4 to 12 years	● Adaptation and negotiation
Low level of preparedness	● Migration objectives could not be reached as planned ● Unexpected economic shocks at destination	● Tangible resources (i.e., few savings)	On average, from 6 months to 3 years	● Households and relatives provide financial support ● Limited resources can be invested as a result of migration experience
No preparedness	● Unexpected family events at home ● No work ● Low Income	● Non-existent	On average, less than 6 months	● Difficult condition at home ● Re migration may be envisaged

**Table 2:** The demographic information of the participants (Source: Authors’ analysis).

Items	Characteristics	N	%
Age	15–25	09	10.71
	25–35	29	34.52
	35–45	31	36.90
	>45	15	17.86
Sex	Male	51	60.71
	Female	33	39.29
	Prefer not to say	00	00.00
Educational Background	Class I – Class V	31	36.90
	Class V – Class X	16	19.04
	HSC	11	13.09
	No Education	26	30.95
Marital Status	Single	08	9.52
	Married	52	61.90
	Widow	16	19.04
	Abandoned	06	7.14
	Divorced	02	2.38
	Prefer not to say	00	00.00
Total Family Members	3	07	8.33
	4	17	16.47
	5	23	19.32
	>5	25	21.00
Returnee's Monthly Income before Pandemic	<3000	19	22.61
	3000–5000	31	36.90
	5000-10,000	20	23.80
	>10,000	09	10.71
Live with Family	Yes	66	78.57
	No	18	21.43
Send Money to Home	Yes	81	96.43
	No	03	3.57

### 4.2 Mapping employability of the migrants

**Fig. 3:**
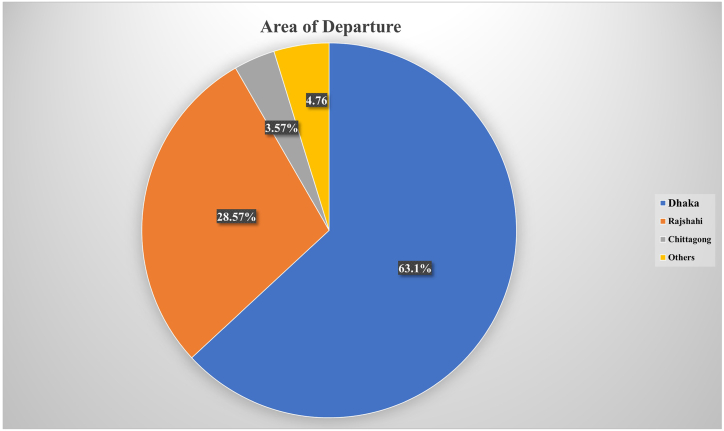
Area of Departure (Source: authors analysis).

As Rangpur is one of the most poverty-stricken areas of Bangladesh, a large number of respondents mostly migrated to Dhaka (63.01%) and Rajshahi (28.57%) (Fig. 3). A small number of migrants (4.76%) have shifted to other parts of the country. Among these migrants, a high percentage of female (32.14%) worked as a housemaid. Similarly, working in garments is also high among female migrants (8.33%) comparing to males (4.77%). Males migrants are mostly found as transport workers (16.67%), rickshaw puller (10.08%), day laborer (9.52%) and street vendor (5.92%). Only a small number of respondents irrespective of their gender are found in formal job sectors (9.52%) after migrating to urban cities (Table 3).

**Table 3:** The Occupation of the participants before Migration (Source: Authors’ analysis).

Item	Characteristics	N	%
Garment Worker	Male	04	4.77
	Female	07	8.33
Rickshaw Puller	Male	12	10.08
	Female	00	00.00
Housemaid	Female	19	32.14
	Male	00	00
Day Labourer	Male	08	9.52
	Female	00	0.00
Construction Worker	Male	04	4.76
	Female	00	00
Transport Worker	Male	14	16.67
	Female	00	00
Street Vendor	Male	05	5.95
	Female	03	2.52
Bank Job	Male	02	2.38
	Female	01	1.19
NGO	Male	03	3.57
	Female	02	2.38

One of the respondents who migrated to the Chattogram division replied,

*“ …. [Neighbour working in garments] suggested that we migrate[struggled to make ends meet cultivating my land and was in debt] at chottogram as the work opportunities and salary for both me and my wife is high while the cost of living is low compared to other parts of the country." (Male Respondent 1, IDI).”*

Most of the respondents’ facing financial difficulties in their place of origin opted to move to areas with higher salaries and lower costs of living highlighting economic factors often drive migration.

Most of the female respondents in their work tenure in urban cities served as a housemaid (32.14%). In terms of male participants, a high percentage (16.67%) were serving as transport workers before the COVID-19 pandemic. A moderate percentage of garment workers (4.77% male and 8.33% female), street vendors (5.95%), day laborers (9.52%), and construction workers (4.76%) also shifted to their native places, temporarily if not permanently. Notably, a small portion of bank job holders (3.57%) and non-government organization (NGO) job holders (5.95%) have also taken part in the reverse migration.

**Fig. 4:**
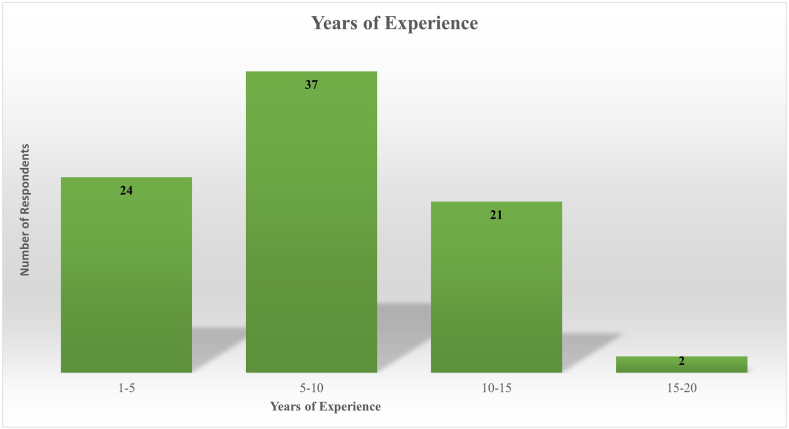
Respondent's years of experience (Source: Authors' analysis).

Fig. 4, followed by Table 3 indicates respondents had 5–10 years of experience of living in urban area 44.05%. On the opposite end, smallest portion (2.38%) of respondents had 15–20 years of experience. In between those, a good portion of respondents had 1–5 years (28.57%) and 10–15 years (2.38%) of experience of living in large, urbanized cities.

Table 4 suggested, economic factors evidently forced 62.28% of households to migrate. Under the economic factor, the urges for higher-income 25.44% motivated the respondents. Along with interest in higher income, poverty (17.54%) and unemployment (19.30%) also fueled the migration. Additionally, the evidence from Table 4 indicated that natural disasters, like Manga (11.40%), floods, and river erosion (14.92%), combined with economic factors, made the migration more accelerated and forceful.

**Table 4:** Factor behind migration of the Returnees (Source: Authors’ analysis).

Item	Characteristics	N	%
Economic Factors	Poverty	20	17.54
	Lower Income	29	25.44
	Unemployment	22	19.30
Natural Disaster	Manga	13	11.40
	Flood and River Erosion	17	14.92
Others		13	11.40

### 4.3 The factor behind reverse migration

It might be assumed that the unplanned migration condition may hinder the people from forming a family. However, a large number of respondents (78.57%) had their family living in the village (Table 2). In terms of assessing the factors behind reverse migration, several viewpoints were found from different Focus Group Discussions (FGD).

“I lost my job due to a nationwide lockdown and the ban on public transport. My wife and I were unable to find other work opportunities, so we decided to return home” (Male Respondent 2, IDI)

Even though the quote was from respondent 2, almost all the respondents suffered economic uncertainty due to nationwide lockdown and ban on public transport. Lose of job due to the pandemic triggered return migration among the individuals and families to secure alternative employment.

“During the hot season, I earn and save more by selling Foler shorbott (fruit juice). But the lockdown left me with no income and cash in hand. I had no work during the lockdown and had no income. So, my savings ran out fast. I was compelled to return to my village at the early stage of lockdown.” (A male respondent of FGD)

During the lockdown, however, I was earning an average of 180 Taka (2 dollars) per day, through which I had to bear the livelihood costs of five members of my family. So, I decided to return to my village because the Boro (winter rice) harvesting season had already started and I could find my income options there.” (Female Respondent 9, IDI)

Adding to the respondent 2, diversified economic impacts of the crisis can be witnessed motivating the individuals to seek out better livelihood opportunities and support systems in their place of origin. In most of the cases, respondents’ lack of income and savings or suffered sharp decrease of income during the lockdown compelled them to return to their village and for the male participate in the Boro harvesting season (agriculture-related work). On the other hand, female are more engaged in household chores with only a few trying to be self-reliant by attaching to handicrafts and rearing cattle highlighting gendered division of labor even during times of crisis.

### 4.4 Returnee's experience and post-return job condition

After retuning, most of them started as daily wage workers (22.62%). One of the respondents mentioned the incapacity of mobilizing resource,

“After returning to the village, I had no work I went to the Jottdar (landlord) of my village for a loan, but he refused as I did not have any tangible assets. My savings have already finished. So, I have decided to go back to Dhaka again to complete my target.” (Female Respondent 2, IDI)

A good percentage of the respondents have started their own business (13.44%) or invested in self-employment (8.33%) (Table 5).

“After coming to the village, I started my new business (a clothing shop) with savings. I have also started fish farming in our pond. I also bought 12 Shotok (decimal) land after returning to the village. Now, I have to depend on the business and fish farming and my income is stable now.” (Male Respondent 6, IDI)

**Table 5:** Job Characteristics of the Returnees in Post-return condition (Source: Authors’ analysis).

Items	N	%
Invest in self-employment	07	8.33
New Business	16	13.44
Fisheries	04	3.36
Cultivation	06	7.14
Depend on Microcredit	07	8.33
Wage laborer	19	22.62
No Income	17	20.24
Others	08	9.52

Another respondent further added:

"The COVID-19 Pandemic has posed a great threat to our progress to be a developed country by 2041. The government has disbursed a fund of Tk5000 crore (5 Billion) for the agriculture sector to boost crop production and overcome the possible coronavirus impact. Assistance such as food, cash transfers, and health financing are provided to poor rural households, including reverse migrants." (Male Respondent 1, KII)

Additionally, approximately 10% of respondents took loans from NGOs with a very low-interest rate.

“*As per the government's announcement, we had stopped collecting weekly installments (Microcredit) till June 30, and we arranged the loan at the minimum interest rate and without any collateral for poor, vulnerable villagers, including reverse migrants. To achieve inclusive development and establish a progressive society, our initiatives resulted in a declining unemployment rate and an increase in their income.” (Male Respondent, 2, KII)*

However, on the opposite side evidence suggests approximately 20% of the respondents have no income right now and solely depended on the savings made during tenure in urban cities.

“After coming to my village, I became unemployed. Now I have to look after my household chores. I earned nearly 12,000 taka during my work tenure. I had cash in hand and did not realize the scarcity during the months of Boishakh, Choittra, and Kartik. Now, I have to solely depend on my savings. I could have spent it on the treatment of my children immediately before COVID-19, but now I cannot because I do not have cash in my hand.”. (A female respondent of FGD)

Respondents are either lacking resources and facing difficulty in securing loans or solely dependent on their savings made during their tenure in urban areas. This highlights the challenges that reverse migrants face in transitioning back to rural areas and the importance of providing support to ensure their livelihoods. Participated returnees were not adequately prepared for the situation they faced upon their return, with almost half of the respondents reporting low levels of preparedness. The government also took initiatives to provide support to reverse migrants, including disbursing funds for the agriculture sector and providing assistance, direct cash transfers, and health financing. Not only that, it requires long term and more comprehensive policies and programs to support the reintegration of reverse migrants into their rural communities.

Moreover, the study found that female returnees were more vulnerable and experience financial crisis than male returnees among participated returnees. It showed that female returnees were not prepared or had low levels of preparedness than man returnees.

### 4.5 Level of preparedness and post pandemic migration trend

**Fig. 5:**
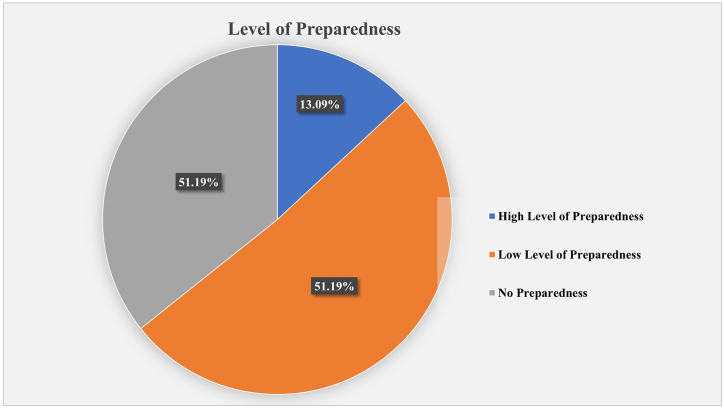
Returnee's level of preparedness.

Fig. 5 shows that the majority of migrants were not well-prepared to deal with the ongoing pandemic.

One of the respondents stated:

“*Before COVID-19, I earned my livelihood by pulling a rickshaw in Dhaka city. But the recent pandemic and imposed lockdown compelled me to return to my village. I had no work. After 3 months, I worked as a day laborer during the planting period of paddy. My savings have already run out. In addition, I had no land or other productive assets in my village. So, I have decided to go back to Dhaka again to complete my target.” (Male Respondent, 5, IDI).*

With that being stated, no preparedness 51.19% and a high level of preparedness 13.09% are also evident among the migrants (Fig. 5).

In conclusion, to address the post-pandemic intention, most of the respondents are interested in the shift to urban areas (46.43%). Living with their families and serving with limited resources, 33.33% have yet to decide whether to stay or return to urban areas (Table 6). However, those migrants who had prior preparedness and mobilized resources would like to permanently settle in a rural area. The strategies suggested by the interviewees and key informants for increasing the living standards of internal reverse migrants are highlighted in Table 7.

**Table 6:** Future Trends of migration of the returnees (Source: authors’ analysis).

Items	N	%
Permanent Settlement	17	20.24
Shift to Urban	39	46.43
Yet not decided	28	33.33

**Table 7:** Suggested strategies by the study participants.

Themes	Categories	Suggested measures
**Employment Generation**	**Access to Credit**	1. Providing migrants with soft financing (below market rate of interest)
	**Access to Nutritious Food**	1. Increasing the number of beneficiaries in “Kajer Binimoye Khaddo” Programme (Food for Work).
		2. Direct incentive measures to social entrepreneurs for nutrition-based activities (Poultries, Fisheries, Agro-farm).
	**Inclusion of marginalized migrants**	1. Frequency enhancement of social safety net programmes for rural migrants
		2. Creating self-employment platform
		3. Stretching humanitarian assistances for pro-poor growth by NGOs
		4. Increase of wage in rural infrastructure and maintenance employment ***“We do not have alternative employment opportunities duing Monga (months of death and disaster), so government should increase the number of beneficiaries in different social safety nets programs.”***
**Gender Specific Measures**	**Capability Enhancement**	1. Educate and empower women through education and skill development programme
		2. Alternative employment opportunities of women migrants
	**Controlling mechanism**	1. Awareness about helpline (i.e. 999)
		2. Strengthen one stop service facilities to minimize violence against women. ***“If women are educated and stop confining themselves in informal jobs, they can be confident and be the bread winners for their family.”***

## 5 Discussion

### 5.1 Status of reverse migrants

The study findings imply that participants associated with informal jobs are prone to reverse migration comparing formal jobholders. Informal jobs are associated with the unorganized sector, in which work, and payment are based on verbal agreements with no contractual obligations on either party. Hossain [28] mentioned in his study that labor-intensive sectors especially those attached to agriculture, export-oriented ready-made garments witnessed significant revenue losses with decreased demand and social distancing tactics [29,30].

According to Hossain [28], a staggering 95% jobs in the agriculture sector are informal. However, service and of industry sector jobs are closing down compared to farming, with 72% and 90%, respectively. The pandemic effect was worse for people who worked in the RMG industry in Bangladesh when demand for Bangladeshi RMG products dropped rapidly in the US and Europe [31]. On this premise, another study found that manufacturing has lost 9.8 million jobs. On the other hand, agricultural jobs have grown by 7.4 million, and workers are back in their villages because of the standstill lockdown [32].

### 5.2 Gender-specific experience

Firstly, as the study findings suggest, men and women returnees alike were subjected to a deplorable scenario during COVID-19, but their experiences were vastly different. Alon et al. [33] predicted that the pandemic, which has prompted critical reflections in a variety of sectors, including gender, education, and work, is anticipated to have a sexist effect. According to the research conducted by Dugarova [34], COVID-19 affects both men and women of all ages. However, it was shown that those between the ages of 60 and 74 had the highest concentration of gender discrepancies with regard to COVID-19 instances.

Secondly, it was evident from this study that, as a result of the COVID-19 crisis, women were forced to make sacrifices in terms of basic needs and to engage in more domestic responsibilities. The crisis poses additional obstacles for women and girls with intersectional characteristics, who face exacerbated disadvantages as a result of the prevalence of unequal structures, power dynamics, and social norms. These interconnected issues restrict their ability to access essential services like healthcare and education and prevent them from actively participating in the decision-making processes that affect their daily existence [34]. In areas like South Asia and Sub-Saharan Africa, where women outnumber males and make up about 90% of the workforce, this scenario proves particularly counterproductive to workers in the informal sector [35].

This study found that female returnees faced severe socio-economic crisis during COVID-19 situation because of low level preparedness or having no preparedness. The findings are consistent with the study of UN Women [36] that migrant workers, particularly women, are more disadvantaged and confront numerous deprivations as a result of poverty [36] and their status as informal workers throughout the pandemic, which also involves them more in domestic chores [37]. Several study findings from Refs. [[38], [39], [40]] indicated that the COVID-19 crisis has supplemented the impact on unpaid care works and the struggle for women to reconcile family and work responsibilities, which leads them to earnings downgrading as well as women's withdrawal from the labor market. According to a survey carried out by Carlson et al. [41] among US parents during the pandemic, it is perceived as the responsibility of mothers to seek out and provide educational material for children. Women's workloads are exacerbated by this additional responsibility, which adds to their already heavy childcare and household responsibilities.

Following reverse migration, women have been relegated to domestic duties, where it has been found in the study that males are more likely than women to shoulder the financial burden. A study conducted by the World Economic Forum ranked Bangladesh 147th in the world for economic participation and opportunity, with women achieving the lowest levels of economic achievement in comparison to males [42].

Thirdly, the findings of the study delve into the state of men's aptitude to conform to the financial downturn and reveal that they carry the heaviest cost of the economic turbulence that has been experienced. In recent years, the country has been one of the best at reducing the gender gap. However, the fallout from COVID-19 has thrown the country's progress into question, especially when it comes to economic participation [42]. The majority of the existing literature has shown how women laborers suffered economically as a result of the COVID-19 outbreak and their awful circumstances after moving to villages. However, the economic stress and suffering of men in villages has been mostly overlooked. The study has explored the missing points in current literature through the lens of men's perspective. UN Women [43] studies revealed that women's ability to endure economic turmoil is lower than that of men. Moreover, the pandemic's long-term and widespread impacts are likely to be different and more severe on women than on men, even though compared with females, men are at a greater risk of being exposed to health-related effects of the COVID-19 pandemic [44].

Fourth, the research documented an instance of marital abuse against women who had returned home during the pandemic. According to the United Nations, “any act of gender-based violence that results or is likely to result in physical, sexual, or mental harm or suffering to women, including threats of such acts, coercion, or arbitrary deprivation of liberty, whether occurring in public or private life” [45,46]. The National Institute of Population Research and Training (NIPORT) recent survey found that around 53% of married women had experienced abuse from their spouses, with 24% reporting experiencing violence in the last year and 60% reporting experiencing violence at some point in their lives. Women of advanced age, those with a large family size, or those from lower socioeconomic groups are more likely to be affected [47,48].

Amid the pandemic, violence against women (VAW) has been on the increase in Bangladesh. Besides, Ain O Salish Kendra (ASK) reported 601 incidents of rape (with the number of cases growing from 76 in April to 94 in May and 174 in June), 107 fatalities of women due to domestic abuse, and 103 cases of sexual abuse, resulting in nine suicides between January and June 2020 [49,50].

### 5.3 Alteration in employment status

The study highlighted that those migrants with organized adequate tangible and intangible resources, along with greater savings and other tangible assets in the community, are well prepared to reverse migration. In addition, the findings suggest that returnees have used their savings as well as their natural and physical capital to start new income-generating ventures in their village. Though their income witnessed downwards trend, this group's socioeconomic standing remained rather stable during the post-reverse migration period. As a consequence, the study showed that they are unlikely to become jobless or underemployed following reverse migration.

The studies of Paraguay and Peru presented similar findings that the returnees were less likely to be unemployed in Paraguay and Peru after their return since they were engaged and invested in agricultural work rather than staying destitute [51,52]. A study finding from a BRAC survey stated that around 47% of Bangladeshi migrant workers have not yet participated in any income-generating activities. In Bangladesh, the study findings contradict a survey finding that 53% of migrant workers have formed their own small businesses or work as day laborers to support themselves financially [53].

As per the findings of the study, those migrants who have a low level of savings and merely tangible or intangible resources have also moved to their village as a result of losing their employment. Semi-skilled workers are referred to as “the workers who have moderately broad knowledge and practical skills and solve regular problems by using simple techniques under supervision but autonomy to a certain degree” [54,55].

A survey on migrant workers in Vietnam [10] showed that approximately 37% returned home with savings, and most of the returnee workers [55] spent their savings to survive during the lockdown. Based on the findings, a study by Dhaka Tribune [56] reported that while 34% of returnees reported having sufficient resources to finance living expenses for up to three months, 33% reported having no savings at all [52].

The study found that migrants with no income and no physical or intangible assets also migrated to their villages owing to the absence of employment opportunities during the countrywide lockdown. FAO [52] explained that returning migrant workers with little resources or no access to land have even fewer options, as rural job opportunities are scarce.

Previous research revealed that 50% of the families obtained a waiver for loan repayment, 8% had to liquidate some assets, 80% increased their debt, and 70% of the sample households made purchases on credit. Farm products was difficult for rural people to sell, while non-farm cottage/business sales fell as a result of weaker demand or supply issues (mostly associated with transportation) [57].

It may cause new social conflicts and disturbances, as well as increased resource demand. In Bangladesh [56], 87% of returnees are unemployed, and 52% require immediate financial assistance, resulting in a fall below the extreme poverty line by 2030 of 160 million people are projected [38].

### 5.4 Lack of service

The study revealed that a concerning lack of services (healthcare, cash assistance, food, trainings) among reverse migrants in the rural areas of Bangladesh. Due to the low savings and quick expense of savings the migrants faced challenges to adapt to the crisis through balancing between life and livelihood. Firstly, they lack financial assistance from the national/local authority which was crucial to cope up the sudden shock of pandemic. Secondly, the lack of financial assistance furthermore constraint taking service of health treatment, food and so on. Previous studies [58,59] focus on the health service especially in the arena of nutrition and hospital facilitation in urban region whereas very few studies [60,61] focuses on the service receiving by the rural minority people in Bangladesh. On this premise, the study documented not only lack of health facilities but also the basic facilities regarding the daily life among the reverse migrants. However, Jahan et al. [55] also focused on the socio-economic reasons for the lack of service facilitation in their findings.

### 5.5 Future trends

The study revealed that while some internal migrants opt to remain in their rural villages and establish sustainable livelihoods, the majority of returnees prefer to return to urban areas due to personal preferences or employment opportunities. The findings is line with The survey on international migrants in the Lao People's Democratic Republic [10] found that 36% of migrant workers planned to leave for a labour destination abroad after the pandemic, 33% planned to stay in the country, and 25% were undecided. Seventy-one percent of Thai returnees surveyed in Cambodia between March and June 2020 expressed a desire to return to Thailand, while only 25% desired to remain. 86% of the people who want to move back want to do so after COVID-19 is over. In addition, Jahan et al. [55] discovered that the majority of returnee employees depleted their savings in order to survive the lockdown. After four months of lockdown, returnee migrants who want to remigrate and return to their old employment confront several obstacles, including financial issues, visa renewal, job contract renewal, plane ticket shortages, and COVID-19 negative certificates.

## 6 Limitations of the study

This analysis had a variety of flaws that must be taken into consideration when analyzing the data. First, the nationwide and area-wise lockdown due to the outbreak of COVID-19 made it impossible for the researchers to cover a wide range of primary sampling units. Second, there were limited pieces of literature in this respective work related to Bangladesh. Subsequently, the researchers have tried to relate to other countries' experiences in the same field. Further, key informant interviews, which were conducted through mobile phone calls, may not provide a comprehensive analysis of the situation. The interviewee may lack openness due to a lack of privacy in phone calls. Finally, the data analysis process might have room to improve for better interpretations.

## 7 Conclusion and recommendations

Migrant workers in the informal sector in developing nations like Bangladesh have been hit particularly hard by the global nightmare that COVID-19 has created overnight. The government of Bangladesh has created economic instability through imposing nationwide and partial lockdowns and holidays, initially from March 26 and extending to May 30, 2020, in different terms. This standard, one-size-fits-all approach to lockdown increased the dire condition of daily laborers. In the densely populated country with a huge density, the consequence has caused an unusual reverse migration, leaving everyone vulnerable to the COVID-19 pandemic. The migrants from informal sectors have a higher tendency to reverse migration than those in formal jobs. Among those migrants, female migrants face negative consequences compared to their counterparts. Most of respondents have experienced a decline in employability, physical capital, and social service. However, the scale of the negative impact varies based on the level of preparedness, which has helped to overcome the employment crisis very fast by investing their tangible and intangible resources in self-employment income-generating activities. Similarly, migrants facing a short-term unemployment crisis overcame the crisis through reinvesting their savings and personal borrowing from relatives in the long run. However, migrants with no savings and no tangible or intangible resources are identified as having no preparedness. They faced a long period of unemployment crisis after post-reverse migration and continuing their hardship due to lack of savings and resources. Comparing the third category to the other two groups, unemployment is more common there.

Practically, this study's findings regarding reverse migrants have significant implications to restore the economic progression Bangladesh was witnessing before COVID-19. This study contributes to the existing body of knowledge in a number of ways, including by highlighting increasing unemployment issues, a lack of physical capital, a lack of preparedness and resources. To minimize the study's limitations and investigate the broader picture of migration issues, mixed-method measures with the triangulation method and a significant number of participants have been selected. The study concludes with a discussion of government and non-government welfare initiatives undertaken to resolve the prevalent problems.

## Funding

This research received no specific grant from any funding agency in the public or commercial agency.

## Author contribution statement

Avijit Saha: conceived and designed the experiments; performed the experiments; analyzed and interpreted the data; contributed reagents, materials, analysis tools or data; wrote the paper.

Arpita Dutta: analyzed and interpreted the data; contributed reagents, materials, analysis tools or data; wrote the paper.

Minhazur Rahman Rezvi: performed the experiments; analyzed and interpreted the data; contributed reagents, materials, analysis tools or data.

Ridwan Islam Sifat: conceived and designed the experiments; performed the experiments; contributed reagents, materials, analysis tools or data.

Dr. Nayeem Sultana: conceived and designed the experiments; contributed reagents, materials, analysis tools or data.

Nuruzzaman: conceived and designed the experiments; wrote the paper.

Mehedi Hasan: contributed reagents, materials, analysis tools or data.

## Data availability statement

No data was used for the research described in the article.

## Additional information

No additional information is available for this paper.

## Declaration of competing interest

The authors declare that they have no known competing financial interests or personal relationships that could have appeared to influence the work reported in this paper.
