# Corrigendum to “Adapting to change: Exploring reverse migration as a coping strategy among internal migrants in Bangladesh” [Heliyon Volume **9**, Issue 9, September 2023, Article e19479]

**DOI:** 10.1016/j.heliyon.2025.e43277

**Published:** 2025-03-29

**Authors:** Avijit Saha, Arpita Dutta, Minhazur Rahman Rezvi, Ridwan Islam Sifat, Nayeem Sultana, Nuruzzaman, Mehedi Hasan

**Affiliations:** a Department of Development Studies, Faculty of Arts and Social Sciences, Bangladesh University of Professionals, Dhaka, 1216, Bangladesh; b Department of Development Studies, Faculty of Social Sciences, University of Dhaka, Dhaka, 1000, Bangladesh; c School of Public Policy, University of Maryland, Baltimore County, Baltimore, MD, 21250, USA; d Department of Environmental Science and Technology, Jashore University of Science and Technology, Jashore, 7408, Bangladesh

In the original published version of this article, the ethics statement was missing information regarding the ethical approval for the study.

The original manuscript showed the ethics statement as:

All the interviews have been carried out with informed written consent. All procedures performed in studies involving human participants followed the ethical standards with the 1964 Helsinki declaration and its later amendments or comparable ethical standards.

This section has been updated to include information regarding the ethics approval. The age range has also been corrected.

The corrected version of the ethics statement can be found below:

All the interviews have been carried out with informed written consent. All procedures performed in studies involving human participants followed the ethical standards with the 1964 Helsinki declaration and its later amendments or comparable ethical standards. Ethics approval was provided by the Department of Development Studies Ethics Committee, Bangladesh University of Professionals, dated 21st July 2020. All participants were over 18 years of age.

The authors apologize for the errors.

## Declaration of competing interest

The authors declare that they have no known competing financial interests or personal relationships that could have appeared to influence the work reported in this paper.

